# Prescribed burning for boreal forest restoration: Evaluating challenges and conservation outcomes

**DOI:** 10.1007/s13280-025-02248-z

**Published:** 2025-09-26

**Authors:** Ellinor Ramberg, Mattias Edman, Gustaf Granath, Jörgen Sjögren, Joachim Strengbom

**Affiliations:** 1 Department of Ecology, Swedish University of Agricultural Science, Box 7044, 75007 Uppsala, Sweden; 2 Department of Natural Science, Design and Sustainable Development, Mid Sweden University, 85170 Sundsvall, Sweden; 3 Evolutionary Biology Centre, Department of Ecology and Genetics, Uppsala University, Norbyvägen 18 D, 75236 Uppsala, Sweden; 4 Department of Wildlife, Fish and Environmental Studies, Swedish University of Agricultural Science, 90183 Umeå, Sweden

**Keywords:** Boreal forest, Conservation, Fire weather, Prescribed fire, Restoration

## Abstract

Prescribed burning is applied as a restoration tool to promote biodiversity in boreal forests. Common objectives include promoting multilayered pine forests, increasing deadwood, and generating fire-scars. However, the extent to which these objectives are achieved and how they relate to weather conditions and stand characteristics remains poorly understood. We surveyed 32 prescribed burns in Sweden to evaluate the outcomes of key conservation objectives and their relationship to weather conditions and stand characteristics. In addition, we compared weather patterns and seasonal timing between prescribed burns and wildfires. We found that conservation objectives were met with large variations between sites but were generally better achieved under drier weather conditions and at sites with higher proportion of spruce. Wildfires occurred under significantly drier conditions and later in the summer than prescribed burns. We conclude that optimizing prescribed burning as a restoration tool requires better alignment between conservation objectives, stand characteristics, and weather conditions.

**Supplementary Information:**

The online version contains supplementary material available at 10.1007/s13280-025-02248-z.

## Introduction

Prescribed burning is used as a management tool in boreal forest ecosystems for a variety of purposes, including reducing fire hazards by lowering fuel loads, as site preparation to promote tree regeneration, and for conserving biodiversity (Weber and Taylor 1992; Ryan et al. 2013; Lindberg et al. 2020). Prescribed burning for biodiversity conservation is applied to restore successional processes (e.g. tree regeneration) and fire-created structures (e.g. deadwood) (Similä and Junninen 2012; Lindberg et al. 2020), as wildfire has in many parts been largely eliminated as a natural disturbance due to effective fire suppression and intensive forest management (Granström 2001; Ryan et al. 2013; Ramberg et al. 2018). In Sweden, prescribed burning is used as a tool to promote biodiversity by county boards as wells as land owners to fulfil forestry certification scheme regulations (Forest Sterwardship Council (FSC) 2020; Life Taiga 2023). Common objectives of these prescribed burns are creation of multi-layered pine forests that are open and sunlit, to increase amounts of deadwood and promote fire-scar formation (Nilsson 2005; Life Taiga 2023).

Creating deadwood and open and sunlit forests requires increased tree mortality. Burn induced mortality is the result of fire intensity (energy released at the flame front), and burn depth (consumption of the ground organic layer) (Ryan 2002; Granström 2005), but also largely depends on tree species adaptions to fire. In Sweden, two coniferous tree species dominate: Scots pine (*Pinus sylvestris*) and Norway spruce (*Picea abies*). Scots Pine are more resistant to fire, characterized by thick bark, deep roots and elevated crowns. Thus, though pine forests are often dry and relatively open and thereby more prone to burning, tree mortality may be variable (Kuuluvainen 2009). Norway spruce has thin bark, shallower roots and lower crowns, characteristics that have been identified as sensitive to fire-induced mortality (Ryan 2002). Spruce forests are denser with moist undergrowth, hampering fire ignition, but in mixed forests, spruce can instead act as ladder fuels, carrying fire to tree crowns (Granström 2005).

Most deadwood forms during or shortly after the fire, but additional input continues as damaged trees die over the following decade (Heikkala et al. 2014; Bär et al. 2019). Charred, sunlit and large diameter deadwood are recognized as important substrates for many species, and are lacking in boreal forests (Siitonen 2001; Jonsson et al. 2016; Hjältén et al. 2018). On damaged, surviving pines fire-scars can form, which is the tree’s physiological reaction to heat induced injuries to the cambium (Baker and Ehle 2001). Such scars are habitats to some specialist species for example *Stephanopachys* species (Wikars 2006), and in the long term the resin-rich wood facilitates the formation of so called kelo wood. Kelo wood is a decay resistant type of deadwood that can develop when old, commonly fire-scarred pine trees die that provide a critical but declining habitat for many threatened lichens and fungi (Niemelä et al. 2002; Larsson Ekström et al. 2023). Tree mortality also increases stand heterogeneity by creating gaps, promoting seedling establishment, and potentially leading to a multilayered forest (Kuuluvainen and Aakala 2011). However, the establishment of pine and deciduous seedlings, such as birch (*Betula sp.)* and aspen (*Populous tremula*), often require exposed mineral soil (Johnstone and Chapin 2006; Gustafsson et al. 2019). Therefore, to meet conservation objectives, prescribed burns must be deep enough to consume much of the organic layer.

For wildfires it is recognized that both weather and vegetation affect fire behaviour, which in turn influences the burn outcome (Ryan et al. 2013; Loudermilk et al. 2022). Longer periods of dry weather reduces the moisture content of the humus layer, thereby increase combustibility, regulating burn depth (Granström 2005). Once a fire has ignited, burn intensity and spread of the fire are determined by temperature, precipitation, wind speed and relative humidity, as well as fuel continuity and amount (Granström 2005; Boby et al. 2023). Vegetation not only acts as fuel, driving fires through combustion, but forest vegetation structure also affects fire behaviour, as tree spatial arrangement and species combinations can affect microclimate and wind speed influencing fire behaviour (Loudermilk et al. 2022). When planning prescribed burning, understanding how stand characteristics and weather conditions may affect fire behaviour is therefore important. In addition, safety and practical issues, such as proximity to water or road access, need to be considered. In Sweden, the Canadian Fire Weather Index (FWI) system has been adopted to assess fire risk (Granström 2005). Both weather conditions and FWI indices are monitored before and during most prescribed burns (Life Taiga 2023). Burns are generally conducted within ‘burn/weather windows’, where the balance between risk and burn viability are deemed reasonable. However, how burning within this window relates to subsequent outcomes of the burns, has rarely been evaluated (see master thesis by (Hermanson 2020)).

The season of burning is also important. Ryan et al. (2013) found that prescribed burns in North American forests are often conducted under higher moisture conditions than wildfires due to limited resources and safety aspects. As a result, these burns may fall short of achieving restoration goals. Though this issue also has been suggested for conservation burns in Sweden (e.g., Granström 2001), thorough assessment is lacking. In a report by the Swedish Civil Contingencies Agency the majority of wildfires between 1996 and 2018 that were larger than 0.5 ha occurred in July (Sjöström and Granstrom 2022). The historic wildfires season is likely to have been similarly timed, in mid- to late-summer, when dry weather conditions coincided with peak lighting activity (Granström 1993).

In this study, we aim to answer some essential questions related to conservation objectives of prescribed burns, with the purpose of improving burn execution. Knowledge on what effects of fire can be achieved when burning within ‘weather windows’ is necessary to set realistic conservation objectives of prescribed burns. Specifically we aim to (1) assess the status of three general conservation objectives of burning: creation of multilayered pine forests, generation of deadwood, and potential formation of fire scars through inventory of 32 sites prescribed burning in Sweden; (2) examine how the status of the three conservation objectives relate to weather conditions and stand characteristics in an attempt to determine key factors that influence prescribed burn effectiveness; and (3) compare the weather conditions of prescribed burns and wildfires to illustrate the difference between the two fire types and provide insight into mechanisms that may affect the ability of prescribed burning to mimic wildfire effects.

## Materials and methods

### Study design and field inventory

**Fig. 1:**
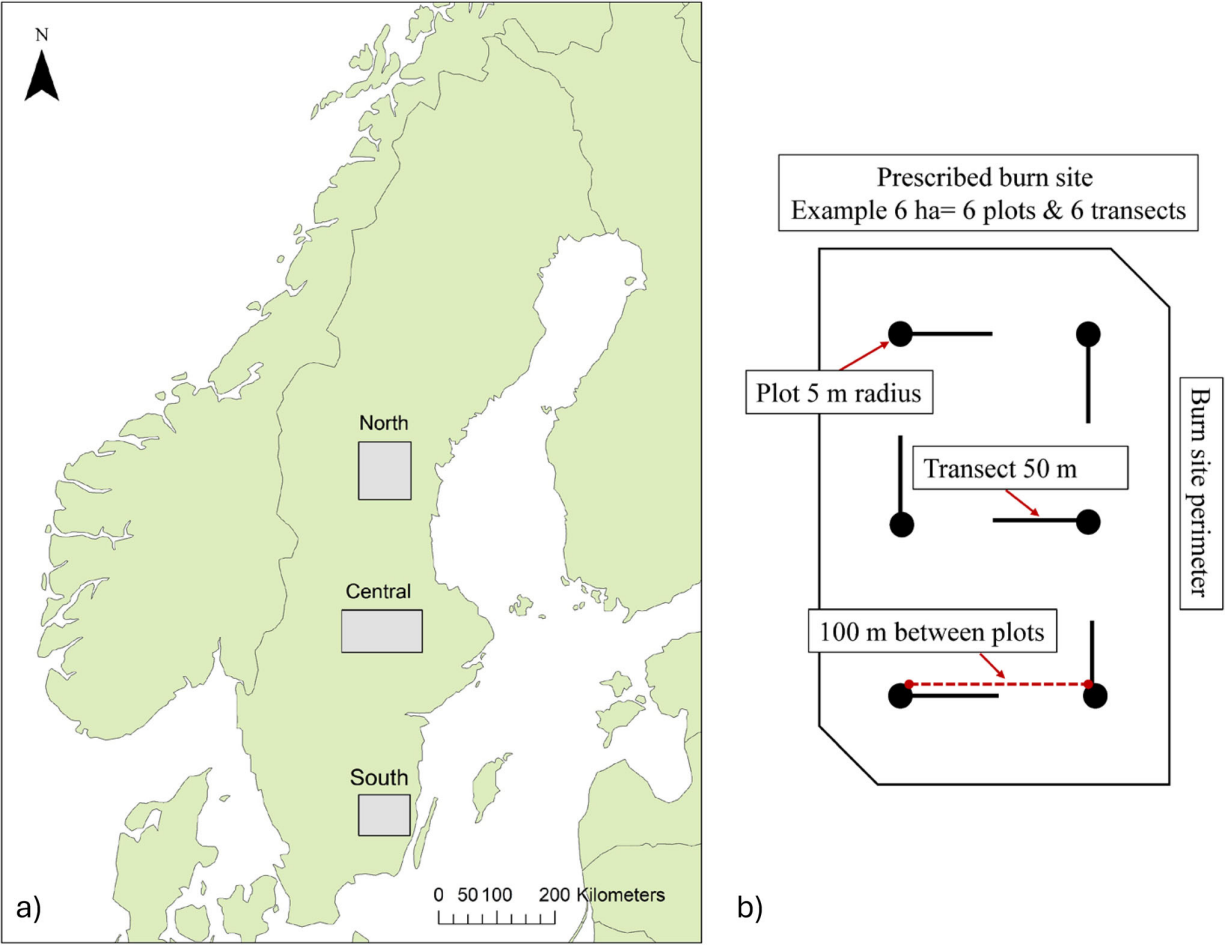
**a** Map showing the three study regions in Sweden and **b** Example of how plots and transects were arranged within a burn site

The study was conducted in Sweden within the middle and southern boreal zones (Ahti et al. 1968). In total, 32 prescribed burns were included (Fig. 1a; Table S1). Sites were chosen based on burn area (≥ 4 ha), burn year (2015–2018, except for two sites burnt in 2014 and 2019, respectively) and logistics (travel distances). All sites were pine-dominated stands within protected areas (nature reserves, national parks or Natura 2000 areas). The sites were part of the EU funded project Life taiga (2015–2020) involving 14 counties that conducted prescribed burns with the aim to ‘benefit biodiversity in taiga forests’ (Life Taiga 2023).

**Fig. 2:**
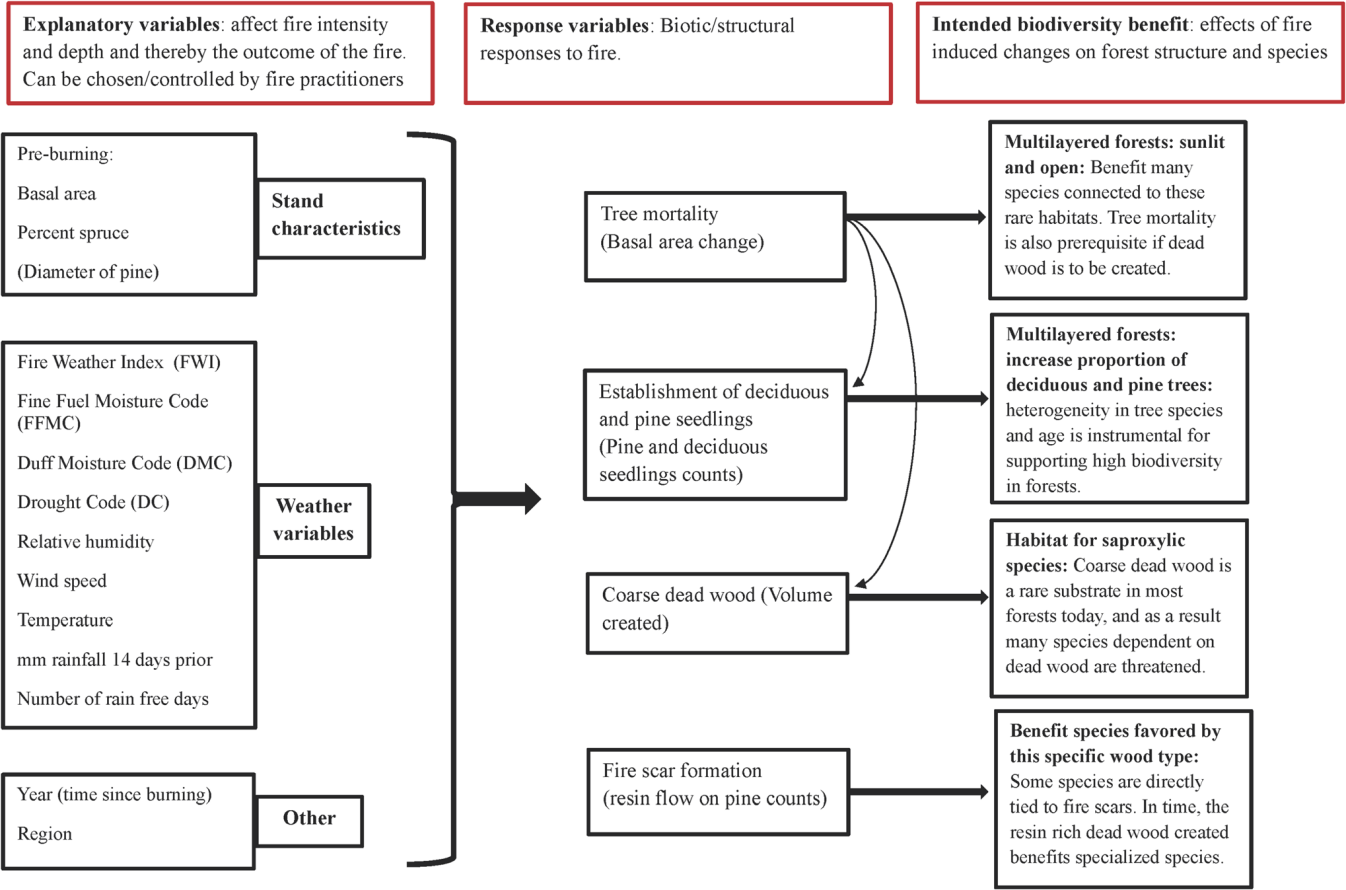
Schematic overview of the explanatory and response variables considered in this study and the intended biodiversity benefit as a result of prescribed burning. Arrows between response variables indicate inter-variable relationships

Field surveys were conducted during the 2020 vegetation period. Site data are summarized in Table 1. Survey plots (5 m radius) were spaced 100 m apart in a grid pattern covering each site, averaging one plot per hectare (Fig. 1b). Tree mortality (change in live basal area) and regeneration (seedling counts) were used to assess the potential for multilayered forest development (Fig. 2). In each plot, all trees (dead and alive) were identified to species level and measured for diameter at breast height (DBH, 130 cm above ground). Dead trees were classified as either pre-fire or fire-related mortality, based on charring, decay stage, and signs of secondary damage (e.g., insect activity). This classification helped reconstruct pre- and post-fire stand structure to evaluate fire effects. Pre-burn stand characteristics, (basal area and mean DBH), were calculated using all trees (DBH ≥ 1 cm), while post-burn characteristics included only living trees. Tree seedlings of all species were systematically counted in each plot to estimate regeneration. To assess fire-scar potential, all living pine trees were examined for resin flow on the lower trunk.

**Table 1:** The variables measured in the plots and transects including details on how they were measured and the motivation behind their inclusion

Variable	Location	Details	Motivation
Tree species	Plot	Alive and dead trees. Dead trees identified by bark or other structures giving indication of species	Describes the tree species distribution both before and after the fire. Spruce mortality is a goal of many burns
DBH of trees	Plot	Diameter at breast height (1.3 m). Measured using a caliper for all trees ≥ 1 cm	Describes the tree size distribution, both dead and live. Also used to calculate basal area (m^2^ ha^−1^) of live and dead trees. A change in basal area after burning gives an indication of tree mortality
Deadwood age	Plot	The standing deadwood within the plot was examined to determine if they had died due to the fire or before. Decay stage, charring, absence of bark and needles, stem breakage and insect tracks were used as indicators	Gives an indication of fire-induced tree mortality
Fire scar formation	Plot	All live pines were scrutinized for signs of resin flow on the outside of the lower tree trunk	Resin flow indicates an injury caused by the fire and over time a fire scar may form. Pines that have survived fire and formed fire scars are often resilient. Many species are associated with fire scars and the resulting deadwood
Tree seedling establishment	Plot	All seedlings per tree species were counted systematically scanning the plot in a zigzag pattern starting from the center. Re-sprouting trees were also included, though they were rare. Height criteria was based on species (but all ≤ 130 cm)	Indication of burn depth as seedlings, especially deciduous species, need a shallow or no humus layer to germinate A goal of burning is often to increase deciduous trees, and seedlings give an indication if this goal will be met
Coarse deadwood standing	Transect	All standing deadwood ≥ 10 cm DBH, within 2.5 m on each side of the transect line. Species noted. Height measured using a Clinometer. Also noted if created before or after burning. The volume of standing deadwood was calculated using the equation for a cylinder based on the height and diameter measurements taken	Used to calculate the volume of deadwood created by the fire. To create deadwood is commonly a goal as many species are dependent on this substrate
Coarse deadwood fallen	Transect	All lying deadwood ≥ 10 cm at DBH was measured at the point crossing the transect. Species was identified if possible. The volume (*V*) of fallen deadwood in transects was calculated using (Van Wagner 1968) equation: ${V=\pi }^{2 }\times \sum_{i=0}^{n}\left(\frac{{D{T}_{i}}^{2}}{8L}\right)$ DT = diameter (in dm) of wood item *i* at transect crossing, L is the transect length (in m)	Used to calculate the volume of deadwood created by the fire

In between the plots we surveyed the amounts of deadwood (DBH ≥ 10 cm) along 50 m transects (Fig. 1b). To minimize the impact of the direction of wind-felled trees direction of the transects were altered between plots. For downed deadwood we measured the diameter at the intersection with the line transect (Van Wagner 1968). Standing deadwood was measured in a 5 m belt along the transect line. Deadwood identified as dead before (following the aforementioned method) burning was used to calculate the pre-burning volumes, and all deadwood was used to estimate the post-burning volumes.

The stand characteristics pre-burning spruce proportion and diameter of pines were calculated using the data collected as described above.

### Weather variables

We used Fire Weather Index (FWI), Fine Fuel Moisture Code (FFMC), Duff Moisture Code (DMC), Drought Code (DC), temperature, wind speed, relative humidity, millimeters of rain 14 days prior to burning and number of rain-free days before burning (details in Table S2) as weather variables. Of our 32 sites, 28 had weather data in their reports from the county administrative boards. Weather data for the remaining four sites (Table S1) were retrieved from the Swedish Civil Contingencies Agency (MSB) database, using historical modelled weather and FWI index values. Additionally, as we wanted to compare the weather conditions between our prescribed burns and wildfires, we extracted data from all wildfires larger than 50 hectares in Sweden between 2012 and 2021 from the MSB database, resulting in 31 wildfires. The minimum size of 50 ha was chosen as these were deemed more likely to follow natural fire patterns (which prescribed burns aim to mimic) compared to the many smaller recorded fires which are often extinguished quickly (Ramberg et al. 2018; Sjöström and Granstrom 2022). Weather and FWI index data on the day these fires started was retrieved from MSB database using the same method described above.

### Data analysis

All statistical analyses were done in R studio in the statistical programming environment R (version 4.3.1, (R Core Team 2024)).

We used paired t-tests to analyse the difference per site before and after prescribed burning in basal area and deadwood volumes (total, pine, spruce, and deciduous) and the mean diameter (DBH) of all trees. Assumptions of equal variance and normality of residuals were assessed, and transformations applied when appropriate.

Each prescribed burning objective was assessed separately with a series of regression models to test for relationships with weather variables and stand characteristics, as well as interactions. We had five response variables: change in basal area, number of seedlings of pine and deciduous trees (birch, aspen, sallow: *Salix Caprea* and rowan: *Sorbus aucuparia*), volume of deadwood and number of potential fire-scars. The explanatory stand characteristics were pre-burning basal area and spruce proportion. For models with fire-scars we also included mean DBH of pine trees as an explanatory variable. The explanatory weather variables were: FWI, DMC, DC, FFMC, relative humidity, wind speed, temperature, mm of rain 14 days before burning, and number of rain free days (Fig. 2). Due to correlations between factors, models were limited to including one response variable, one stand variable, and one weather variable. We also examined if the region and time since burning had an influence on the response variables. Years were sorted into three classes (2014–2015, 2016–2017, 2018–2019) as the years 2014 and 2019 only had one burn each. All explanatory variables were standardized prior to model fitting. A very high DMC in one site was deemed unreliable and was thereby excluded in all analyses with DMC. Model assumptions and fit was assessed using diagnostic plots (R packages: DHARMa & betareg, (Cribari-Neto and Zeileis 2010; Hartig 2022)).

Beta regression models were used for basal area change as it is a proportion (betareg package, (Cribari-Neto and Zeileis 2010)). Likelihood ratio tests were utlilized to assess each explanatory variable´s effect on the response (lmtest package, (Zeileis and Hothorn 2002)). To test for differences between regions we used post-hoc Tukey tests (emmeans package, (Lenth 2024)). To account for overdispersion associated with count response variables (seedlings and fire scars), we used negative binomial generalized linear models (GLMs) (MASS package, (Venables and Ripley 2002)). As several zeros occurred in the deciduous seedling data, we checked if negative binomial GLMs were under-fitting zeros (performance package, (Lüdecke et al. 2021)). If zero-inflation was found we used zero-inflated negative binomial models (pscl package, (Zeileis et al. 2008)). Two sites had very high deciduous seedling counts (> 6000), which disproportionately affected the models, thus all models were run with and without these two sites. For the volume of deadwood linear models (LMs) with a Gaussian error distribution were utilized. Volume of deadwood was log-transformed to fit model assumptions of equal error variance and normality.

To examine how the weather conditions differed between the prescribed burns and wildfires during the same period we conducted Welch two sample t-tests for FWI, FFMC, DMC, DC, relative humidity, temperature, and wind speed. We illustrated the differences in fire season by comparing the Julian dates of prescribed burns with wildfires in a histogram and density plot.

## Results

### Multi-layered pine forests, sunlit and open

**Fig. 3:**
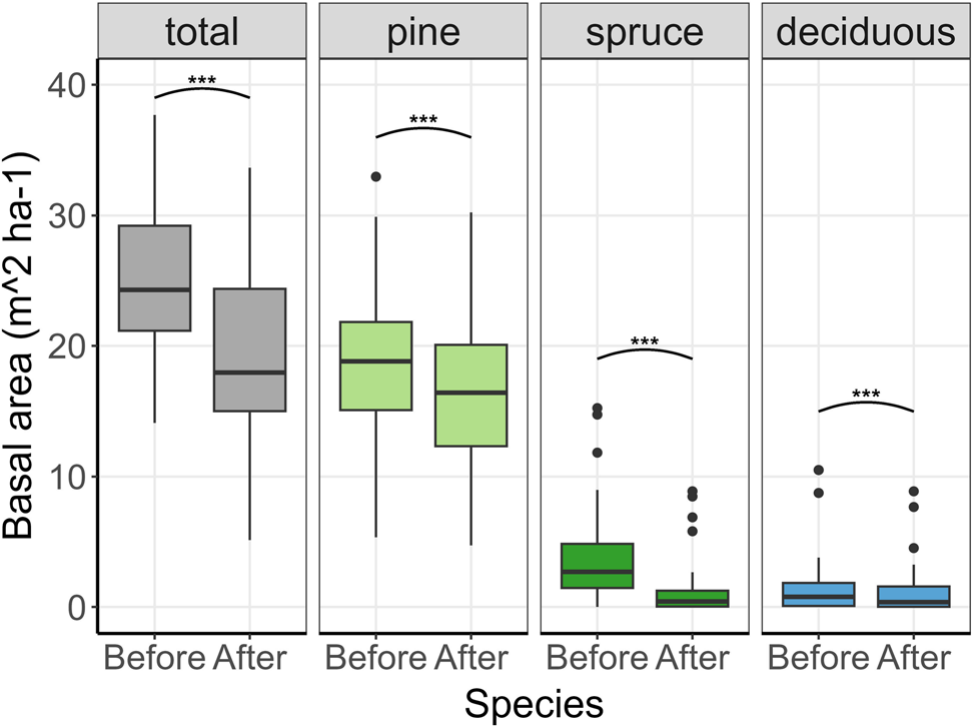
Boxplots of basal area per site (*N* = 32) before and after prescribed burning for the total, pine, spruce and deciduous (birch, aspen, sallow, rowan) trees. Whiskers correspond to ± 1.5* Inter quartile range. *** = *p* < 0.001 for a paired t-test

The basal area of living trees of pine, spruce, and deciduous species all decreased after burning (Fig. 3; Table 2). Mean tree diameter (DBH) increased from 13.13 ± 2.24 cm (mean ± SD) before burning to 20.22 ± 4.69 cm after (t_31_ = 14.5, *p* < 0.001, Table S3).

**Table 2:** The mean and standard deviation of the basal area (m^2^ ha^−1^) of the total, pine, spruce and deciduous trees before and after burning and the results of paired t-tests. Spruce and deciduous basal area were square root transformed to achieve equal error variance and normality

Tree species	Basal area before mean (SD)	Basal area after mean (SD)	t-value	df	*p* value
Total	24.9 (5.8)	19.2 (6.6)	7.1	31	**< 0.001**
Pine	19.1 (5.5)	16.4 (6.3)	4.2	31	**< 0.001**
Spruce	4.1 (4.1)	1.5 (2.5)	7.7	31	**< 0.001**
Deciduous	1.7 (2.8)	1.3 (2.1)	4.2	31	**< 0.001**

Region was important for explaining the variation in basal area change as it significantly improved the fit of the model (basal area change ~ year + region) compared to the simpler model (basal area change ~ year) (χ^2^ = 11.4, df = 2, *p* = 0.003, all model coefficients in the results section can be found in Table S4). Post hoc Tukey test showed smaller reductions in the central region (13% ± 8%) compared to the northern (22% ± 13%, *p* = 0.04) and southern regions (33% ± 17%, *p* = 0.01, All post-hoc coefficients can be found in Table S5). Time since burning did not significantly improve model fit (χ^2^ = 4.9, df = 2, *p* = 0.08).

The change in basal area was positively correlated with pre-burn spruce proportion, with spruce proportion significantly improving the beta regression model fit (basal area change ~ spruce proportion; χ^2^ = 18.9, df = 3, *p* = 0.03).

### Multi-layered pine forests, increase proportion of pine and deciduous trees

Across sites and region, mean seedling abundance was 1744 (SD ± 2252) per ha for pine and 1430 (SD ± 2685) for deciduous trees, with high variability among sites (Fig. S1). Pine seedling abundance differed significantly among regions, (χ^2^ = 14.7, df = 2, *p* < 0.001), but not with time since burning (χ^2^ = 0.05, df = 2, *p* = 0.97), and there was no interaction between the two variables (χ^2^ = 2.7, df = 3, *p* = 0.44). Post hoc Tukey test revealed that the northern region (Mean ± SD = 308.7 ± 273.8) had fewer pine seedlings compared to the central (2238.9 ± 1728.6, *p* < 0.001), and southern regions (986.6 ± 1221.5, *p* = 0.03). The only weather variable that was associated with pine seedling abundance was FWI, (χ^2^ = 9.5, df = 1, *p* = 0.002), suggesting reduced establishment under high fire intensity.

Deciduous seedling numbers also varied among regions (χ^2^ = 8.76, df = 2, *p* = 0.01), but not with time since burning (χ^2^ = 0.17, df = 2, *p* = 0.92). Post hoc Tukey test revealed that the southern region (3930.3 ± 4595.7) had higher seedlings counts compared to the central (624.1 ± 697.7, *p* = 0.02), and northern regions (539.9 ± 481.6, *p* = 0.03). Without two extreme sites (N = 29) analyses indicated that deciduous seedlings increased with increasing proportion of spruce pre-burning (estimate = 0.68,* p* = 0.001) and DMC (estimate = 0.81,* p* = 0.007). There was also a significant interaction between DMC and pre-burn spruce proportion (estimate = -0.60,* p* = 0.001), with a stronger positive influence of DMC under low than high spruce proportions. Including the two sites with extreme deciduous seedling counts (N = 31) removed these effects (Table S4).

### Creation of deadwood

**Fig. 4:**
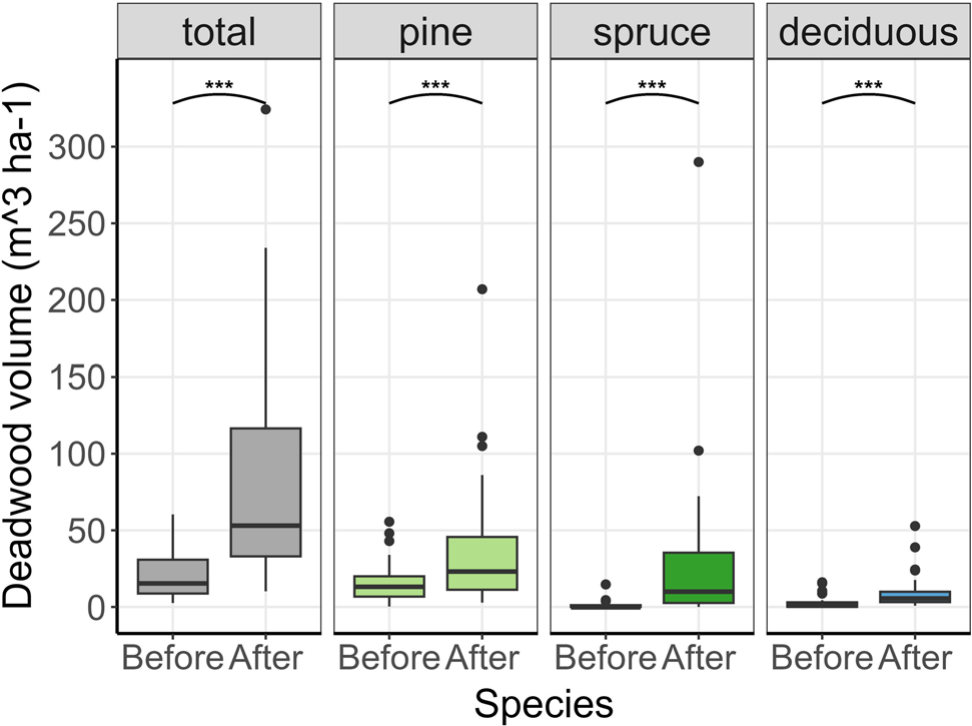
Boxplots of deadwood (standing and downed, DBH > 10 cm) volume per site (*N* = 32) before and after prescribed burning for the total, pine spruce and deciduous (birch, aspen, sallow, rowan*,*) trees. Whiskers correspond to ± 1.5* Inter quartile range. *** = *p* < 0.001 for a paired t-test

Burning significantly increased the volume (m^3^ ha^−1^) of pine, spruce, and deciduous deadwood (Fig. 4; Table 3). Post-burn deadwood (total DBH ≥ 10 cm) was on average more than threefold higher than estimated pre-burn volumes (Fig. 4; Table 3). The variation among sites was, however, large ranging from an increase of only 2.5 to as high as 283 m^3^ ha^−1^.

**Table 3:** The mean and standard deviation of the mean deadwood volume (m^3^ ha^−1^) for the total, pine, spruce and deciduous trees before and after burning and the results of paired t-tests. All deadwood means were log transformed to achieve equal error variance and normality

Tree species	Deadwood volume before mean (SD)	Deadwood volume after mean (SD)	t-value	df	*p* value
Total	20.2 (15.3)	75.6 (67.3)	10.8	31	**< 0.001**
Pine	15.8 (13.3)	36.8 (41.7)	− 4.6	31	**< 0.001**
Spruce	1.6 (3.6)	29.4 (53.6)	− 9.2	31	**< 0.001**
Deciduous	2.8 (4.4)	9.4 (11.4)	− 10.2	31	**< 0.001**

Though there was a trend towards increasing deadwood volumes with time since burning, the effects was not statistically significant (F_2,24_ = 2.52, *p* = 0.10). Neither was there a difference among regions (F_2,24_ = 2.77, *p* = 0.08), nor an interaction between region and time since burning (F_3,24_ = 1.87, *p* = 0.66).

Deadwood creation increased with pre-burn basal area (F_1,28_ = 3.98, *p* = 0.05) and pre-burn spruce proportion (F_1,28_ = 11.98, *p* = 0.002). It was also positively related to the weather variables FWI and DC (F_1,30_ = 7.66, *p* = 0.01), increasing at high FWI (F_1,30_ = 5.79, *p* = 0.02) and DC (F_1,30_ = 7.66, *p* = 0.01),

### Promotion of fire-scars

The proportion of pines with resin flow after burning was on average 46% (SD ± 25) (per hectare: Mean ± SD = 195, ± 176, (Fig. S2)). There was, however, a considerable variation among sites, ranging from no scars to 89%. There was no difference in the number of potential fire-scars among regions, or in relation to time since burning, pre-burn stand characteristics, or with weather variables. However, the number of potential fire-scars decreased with increasing mean DBH of pines (χ^2^ = 8.77, df = 1, *p* = 0.003).

### Fire weather

**Fig. 5:**
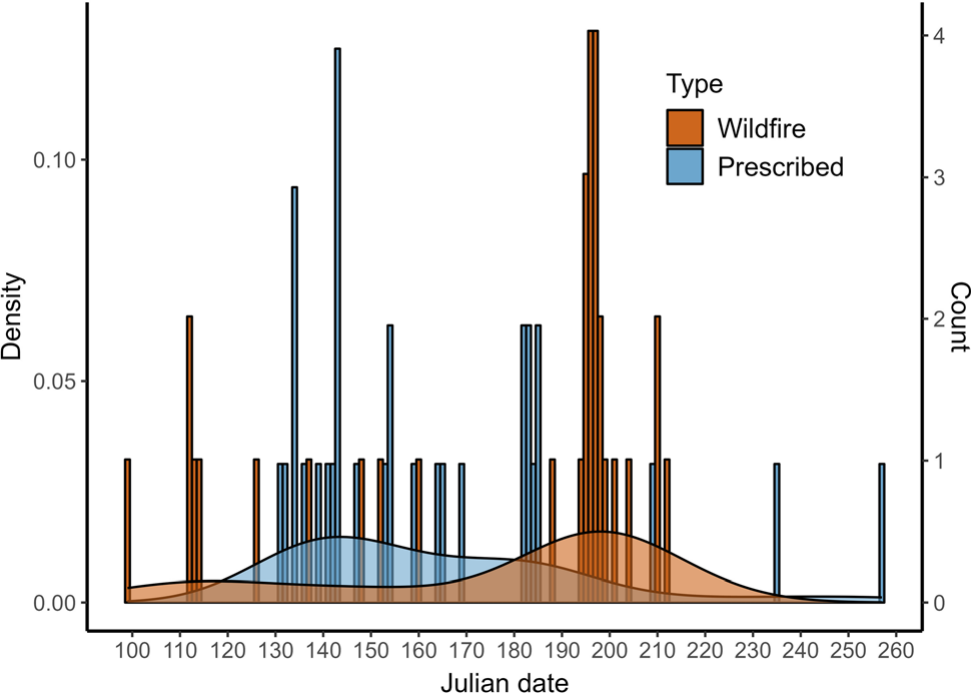
Histogram and density plot showing the distribution of the Julian dates of prescribed burns in this study (*N* = 32) and wildfires (*N* = 31) which occurred during the same years

Prescribed burns were carried out earlier in the year compared to wildfires (Fig. 5). All tested weather variables, except for FFMC, differed significantly between wildfires and prescribed burns included in our comparison (Table 4). Specifically, wildfires occurred at higher temperatures, wind speeds, FWI, DMC and DC and at lower relative humidity compared to prescribed burns.

**Table 4:** The mean and standard deviation of weather variables per fire type (wildfires (*N* = 31) vs .prescribed burns (*N* = 32)) and the results of Welch two-sample t-test

Weather variable	Wildfires mean (sd)	Prescribed fires mean (sd)	t	df	*p* value
Temperature	24.8 (4.1)	20.8 (4.4)	− 3.8	60.9	**< 0.001**
Relative humidity	33.2 (8.5)	40.4 (11.3)	2.9	57.4	**< 0.001**
Wind speed	3.4 (1.9)	2.5 (1.4)	− 2.1	55.9	**0.04**
FFMC	90.4 (2.8)	89.9 (2.4)	− 0.8	69.2	0.44
DMC	65.3 (29.1)	44.4 (14.5)	− 3.6	43.8	**< 0.001**
DC	288.3 (127.8)	181.5 (79.8)	− 3.9	50.1	**< 0.001**
FWI	25.8 (10.6)	18.3 (5.4)	− 3.5	44.2	**< 0.001**

## Discussion

### Multi-layered pine forests: Sunlit and open

Overall, burning reduced the basal area, although with large variation among sites. Spruce mortality was particularly high, as expected. Changes in basal area were predicted by pre-burn spruce proportion, demonstrating that the likelihood of achieving more open and sunlit environments is largely depended on initial stand composition. Regional variation likely reflects the burn practitioner's site selection, highlighting the importance of aligning site variables with burn objectives, as also emphasized in other European studies of prescribed burning (Fernandes et al. 2013; Lindberg et al. 2020).

Mean tree diameter increased after burning, indicating higher mortality among small-diameter trees, consistent with Linder et al. (1998) and Sidoroff et al. (2007). Bark thickness increases with tree age, which protects the cambium of older trees from fatal damage (Sidoroff et al. 2007). Low-intensity prescribed burns primarily kill small trees, limiting the creation of coarse deadwood, which is more valuable for saproxylic species (Kuuluvainen and Aakala 2011). This size-biased mortality reduces structural heterogeneity, potentially contradicting biodiversity goals in managed boreal forests already lacking structural complexity (Hekkala et al. 2023). However, recurrent fires may restore heterogeneity through varied age-cohorts and enhance landscape-level heterogeneity.

### Multi-layered pine forests: Increase proportion of pine and deciduous trees

Whilst some burns resulted in relatively high seedling establishment, densities varied greatly among sites. We recorded an average of 1430 deciduous seedlings per hectare, which is substantially lower than what is common after wildfires. Gustafsson et al. (2019) reported densities in the range of 11,000 to 17,000 two years after a wildfire. However, our abundances, and the variation between sites, align with previous records from prescribed burns (Fredriksson et al. 2023).

Seedling establishment was positively correlated with Duff Moisture Code (DMC), which reflects dryness in the surface organic layer. Low DMC values, indicating moist conditions, likely resulted in shallow burn depth and poor establishment, consistent with studies showing few seedlings where more than 2 cm humus remained (Johnstone and Chapin 2006; Gustafsson et al. 2019). Two sites had very high deciduous seedling counts, likely due to aspen root suckering, a vegetative regeneration which is facilitated by disturbance, but not dependent on burn depth (Rogers et al. 2020). Surprisingly, density of pine seedlings was negatively correlated with Fire Weather Index (FWI). While high FWI promotes intense fires, it may leave behind a charred, hydrophobic ground layer that hinders germination (Johnstone and Chapin 2006). We also found that deciduous seedling density increased with the pre-burn spruce proportion, likely because spruce stands often occur on mesic sites, which also favour establishment of deciduous seedlings (Latva-Karjanmaa et al. 2006).

Our results confirm that dry surface conditions are important for pine and deciduous seedling establishment. However, deep burns require more effort to secure boundaries and prolonged post-burn monitoring, as smouldering fire can reignite under favourable conditions. Balancing cost and safety with conservation goals is essential, and mechanical humus removal may be an alternative for promoting seedling establishment.

### Creation of deadwood

Deadwood is essential for saproxylic species, yet many forests lack sufficient amounts (Siitonen 2001; Kyaschenko et al. 2022). Burning generally increased coarse deadwood volumes across all tree species, though with high site-level variation. While our study did not explicitly assess deadwood diversity, we observed that only a few sites produced substantial volumes of pine, spruce, and deciduous deadwood. This may limit the conservation value, as a variety of dead wood types is important for maintaining biodiversity (Berglund et al. 2011; Seibold et al. 2016). Similar increases in deadwood following prescribed burning have been documented in North American forests (Stephens and Moghaddas 2005; Harrod et al. 2009). Additionally, studies in Fennoscandian conifer forests have shown that prescribed burning can also increase structural diversity in deadwood, highlighting the broad applicability of fire as a tool for deadwood creation (e.g. Shorohova et al. 2024).

As expected, deadwood formation was positively correlated with the FWI values. High FWI values indicate conditions favourable for high-intensity fires, which typically cause greater tree mortality and thus more deadwood (Ryan 2002; Granström 2005). We also found a positive relationship between deadwood volume and Drought Code (DC), highlighting the importance of dryness in the deeper organic layer. In Sweden, higher DC values usually occur late in summer (Granström 2005). A dry organic layer can lead to deeper burns and increase tree mortality (Bär et al. 2019). Relationships between several of the FWI components and deadwood volume and seedling counts suggest that the FWI system is a valuable tool not only for planning safe burns but also for predicting the conservation outcomes of prescribed burns.

Both pre-burn basal area and spruce proportion were also positively associated with formation of deadwood. Spruce mortality was particularly high, and sites with greater initial spruce proportion had more potential to produce deadwood. Additionally, spruce likely acted as ladder fuel (Granström 2005), increasing fire intensity around pine trees and contributing to additional mortality. The high basal area at many sites was primarily due to dense stands of younger trees, which are more susceptible to fire-induced mortality, and thus more likely to contribute to deadwood volumes.

In several sites we noted beetle exit holes on dead trees, indicating presence of wood-boring beetles. Post-fire mortality is commonly an extended process, with fire-damaged trees dying gradually due to interaction with secondary disturbances, such as insect attacks and fungi (Bär et al. 2019). However, we found no effect of time since burning on total deadwood volume. This likely reflects that some burns, especially early in the time series, caused minimal initial damage, which may obscure patterns of deadwood accumulation across sites over time.

### Promotion of fire-scars

The potential for fire-scar formation varied greatly among sites, which confirms earlier findings by Piha et al. (2013). Lack of scarring on a living tree is either due to either low fire intensity, or due to absence of fire near the tree (Baker and Ehle 2001), which may explain both variability and the low number of scarred trees in some of our sites. Our finding that fire-scars were more common on smaller, younger pine trees confirms that higher-intensity fires are required to scar older pines with thicker bark. Formation of fire-scars is important in the short-term for maintaining populations of species that reproduce in the injured tissue (Wikars 2006), and over a longer time span, fire-scars contribute to the development of habitats for species such as the lichen *Hertelidea botryosa*, which depend on the resulting slowly formed deadwood (Larsson Ekström et al. 2023).

### Fire weather and its implications

With the exception of FFMC, all tested weather variables differed between wildfires and prescribed burns. FFMC values were similar, as moist fine fuel layers cannot sustain fire (Granström 2005; Lindberg et al. 2021). The differences in other weather variables confirm that prescribed fires are typically conducted under different conditions than wildfires. Prescribed burns also occurred earlier in the season compared to wildfires. While these seasonal and weather differences are familiar to practitioners, emphasizing them can help shape more realistic conservation goals. For example, early-season burns are unlikely to coincide with dry ground conditions (DMC and DC), making high deciduous seedling establishment an unrealistic objective. Conducting prescribed burns under wildfire-like conditions is often unfeasible for safety reasons, a limitation also emphasized in North American fire management, where safety constraints can reduce the effectiveness of prescribed fire (Ryan et al. 2013). However, one strategy could be to categorize prescribed burns into early and late summer types, aligning conservation objectives accordingly, along with adjusted burning techniques and safety measures.

## Conclusions

Prescribed burning as a restoration tool has the potential to create forest structures and restore successional processes important for the conservation of biodiversity. However, our study reveals substantial variation between sites in achieving key conservation objectives, such as creating multilayered pine forests, producing deadwood, and forming fire-scars, highlighting the need to adjust either the objectives or the burn execution. Both location and timing of burns can be controlled. While safety aspects are imperative to consider, it is evident that further efforts are needed to reach the targeted conservation outcomes. Burn objectives and expectations can be tailored to weather and season, allowing for different burn types. Forest composition and structure, particularly the proportion of spruce, significantly impact burning outcomes and the achievement of various restoration goals. Rather than being an all-encompassing restoration method, prescribed burning should be guided by clearly defined objectives that are adapted to each site’s specific characteristics and prevailing weather conditions.

## Supplementary Information

Below is the link to the electronic supplementary material.

Supplementary file1 (PDF 808 kb)

## Data Availability

Data is available at zenodo 10.5281/zenodo.17053083.
