# Supplementary material for: Prescribed burning for boreal forest restoration: Evaluating challenges and conservation outcomes
Source: Ambio. 2025 Sep 26;55(3):608–19. doi: 10.1007/s13280-025-02248-z (PMC12868470; doi:10.1007/s13280-025-02248-z)
Supplement: Supplementary file 1 — Supplementary file1 (PDF 808 kb) [file 13280_2025_2248_MOESM1_ESM.pdf]

AMBIO

Supplementary Information

This supplementary information has not been peer reviewed.

Title: **Prescribed burning for boreal forest restoration: Evaluating challenges and conservation outcomes**

Authors: Ellinor Ramberg*, Mattias Edman, Gustaf Granath, Jörgen Sjögren, Joachim Strengbom

*Corresponding author: ellinorramberg@hotmail.com

Table S1. Characteristics of the prescribed fire sites included in this study, including the site name, region, county, protection type, the area burnt and the year that the sites were burnt. * indicates sites with incomplete weather data.

**Name**	**Region**	**County**	**Protection type**	**Area burnt (ha)**	**Year burnt**
Lappland A	Central	Västmanland	Nature reserve	20.2	2016
Lappland B	Central	Västmanland	Nature reserve	16.7	2014
Öberget (Färnebofjärden national park)	Central	Västmanland	National park	40.5	2018
Stora Flyten A	Central	Västmanland	Nature reserve	9.9	2017
Stora Flyten B*	Central	Västmanland	Nature reserve	13	2018
Ställbergsmossen A*	Central	Örebro	Nature reserve	16	2015
Västeråsmossen A*	Central	Örebro	Nature reserve	27.5	2015
Römyren (Nittälven) *	Central	Örebro	Nature reserve	26.1	2016
Brattforsheden	Central	Värmland	Nature reserve	9.2	2015
Fräkensjömyrarna A	Central	Värmland	Nature reserve	24	2019
Silmamossen V	Central	Dalarna	Natura 2000	10.6	2018
Silmamossen Ö	Central	Dalarna	Natura 2000	12.1	2018
Fux-Andersknallrna	Central	Dalarna	Nature reserve	30.9	2017
Haftahedarna C	Central	Dalarna	Natura 2000	4	2017
Haftahedarna N	Central	Dalarna	Natura 2000	4.7	2018
Haftahedarna S	Central	Dalarna	Natura 2000	4.5	2017
Flistersjön (Helvetesbrännan)	North	Västernorrland	Nature reserve	31	2015
Åkroken (Helvetesbrännan)	North	Västernorrland	Nature reserve	14	2015
Bastunäset S (Jämtgaveln)	North	Västernorrland	Nature reserve	22	2018
Bodmyran (Jämtgaveln)	North	Västernorrland	Nature reserve	40	2018
Stormyran Lommyran	North	Västernorrland	Nature reserve	28	2018
Ensjölokarna	North	Gävleborg	Nature reserve	12	2018
Hagåsen	North	Gävleborg	Nature reserve	19	2018
Stensjön	North	Gävleborg	Nature reserve	9	2018

Påsetegen (Allgunnen)	South	Kalmar	Nature reserve	6.7	2015
Krogölen (Allgunnen)	South	Kalmar	Nature reserve	16.5	2016
Stora och Lilla Fly	South	Jönköping	Nature reserve	17.6	2016
Stolpaberg Ö	South	Jönköping	Nature reserve	17.4	2015
Klocknaberg S (Storasjö)	South	Kronoberg	Nature reserve	16.3	2018
Klocknaberg N (Storasjö)	South	Kronoberg	Nature reserve	19.2	2018
Tonaberg (Storasjö)	South	Kronoberg	Nature reserve	19.5	2016
Singelstorps fly	South	Kronoberg	Nature reserve	27.1	2016

Table S2. All fire weather index and weather variables used in this study, their definition/unit and effect on fire behavior (Van Wagner 1987; Granström & Schimmel 1998; Granström 2005; Boby et al. 2023).

**Weather variable**	**Definition/unit**	**Effect on fire behavior**
FWI (Fire Weather Index)	The intensity of the spreading fire measured as energy output per unit length of the fire front. Calculated from a combination of Initial Spread Index (ISI), where FFMC and wind are combined to represent the rate of spread of fire, and Buildup Index (BUI) which is a combination of DMC and DC representing the available fuel buildup.	High FWI can either be a result of high wind speeds or dry conditions or a combination. Depending on which conditions that the FWI is based on, high FWI can either affect burn depth (DMC and DC high), or burn intensity (high FFMC and wind speed), or both.
FFMC (Fine Fuel Moisture Index)	The moisture content of fine fuels (moss, leaf litter). Possible range is 0-101 with high values representing low moisture content. Over 75 is needed for a fire to spread. Based on rainfall, relative humidity and temperature.	A high FFMC indicates a dry surface fuel layer and the potential therefore for successful ignition and spread of fire. At low wind speeds and high ground moisture levels this type of fire would move along the surface with low intensity and depth.
DMC (Duff Moisture Code)	The moisture content in the upper ground layer (humus or duff layer). The range starts at 0 and is open ended, high values represent low moisture content. The range in Sweden is between 0-150. Over 60 indicates a dry humus layer. Based on rainfall, relative humidity and temperature.	High DMC indicates the possibility for higher burn depth to be reached.

DC (Drought Code)	The moisture content in the deeper, more compact organic ground layer. A low value indicates moist conditions and high values dry conditions. Has no upper limit but is seldom higher than 600 in Sweden. Based on rainfall and temperature.	High DC indicates the possibility for deeper burn depth to be reached.
Air temperature	Measured in Degrees Celsius<br>Optimal prescribed fire conditions are intermediate temperatures.<br><30 °C is usually set as a max.	Affects both evaporation rates so fuels dry out faster at higher temperatures, and heats up fuels so less energy is needed for the fire to ignite them. Affects both burn intensity and depth.
Wind speed	Measured in Meters per second<br>Under approximately 6 m/s is considered safe for prescribed burning.	Can shift abruptly in strength and direction, causing variation in fire intensity. With increasing wind speed the flames are at a narrower angel to the ground, heating fuels and igniting them faster. Also increases evaporation and drying out of fuels prior to burning.
Relative humidity (RH)	Measured in Percent<br>For prescribed burning no lower than the air temperature is recommended.<br>The moisture content threshold in fine fuels for fire to burn is around 22-25% which corresponds to 80-90% RH.<br>For fire to spread uninterruptedly a fine fuel	Has a direct impact on the moisture content of surface fine fuels (moss, lichen, litter) and is thereby the most important factor in the drying of them. Thereby RH has a large impact on fire behavior. Varies throughout the day, increasing in the evening when air temperatures drop.

	moisture content of 5-10%, corresponding to RH 20-30 %, is needed.	
Mm rain 14 days prior	Measured in mm.<br>Generally, burns are not conducted on days when rain is predicted, and preferably, no rain should fall a few days prior to burning.	After a rainfall event that trickles down through surface fuels, and depending on the vegetation structure, the ground layers can take up to at least a 14 days to dry out sufficiently for fire to ignite. How much rain falls in a period leading up the burn, has implications for the moisture levels in the ground layers. How dry the ground layers are impacts burn depth.
Number of rain free days	Count	See above.

Table S3. Results of welch paired t-tests for the difference before and after burning on the mean total diameter at breast height (DBH) and mean pine DBH per site. Statistically significant results are written in bold.

DBH (cm)	Before mean (SD)	After mean (SD)	t	df	*p*-value
Mean total (log)	13.13 (2.24)	20.22 (4.69)	14.5	31	**<0.001**
Mean pine	20.09 (5.79)	23.41 (5.04)	6.58	31	**<0.001**

Table S4. Model coefficients for models reported in the results section of the main document. P-values < 0.05 are in bold.

Response variable	Explanatory	Estimate	SE	z value	p value
**Basal area change (beta regression model)**	Intercept	-1.63	0.33	-4.98	**<0.001**
	Region N	1.04	0.38	2.72	**0.006**
	Region S	1.14	0.36	3.19	**0.002**
	Time (2016,2017)	-0.25	0.38	-0.67	0.50
	Time (2018,2019)	-0.91	0.43	-2.15	**0.03**
**Basal area change (beta regression model)**	Intercept	-2.06	0.25	-8.22	**<0.001**
	Region N	0.85	0.37	2.29	**0.02**
	Region S	1.37	0.35	3.93	**<0.001**
**Basal area change (beta regression model)**	Intercept	-1.26	0.35	-3.65	**<0.001**
	Time (2016,2017)	0.16	0.41	0.40	0.69
	Time (2018,2019)	-0.77	0.45	-1.70	0.09
**Basal area change (beta regression model)**	Intercept	-1.25	0.18	-6.89	**<0.001**
	Spruce proportion	0.36	0.16	4.11	**0.03**
**Pine seedlings count (Negative binomial GLM)**	Intercept	7.91	0.53	14.89	**<0.001**
	Region N	-2.99	0.92	-3.24	**0.004**
	Region S	-0.59	1.19	-0.49	0.62
	Time (2016,2017)	-0.15	0.68	-0.22	0.83
	Time (2018,2019)	-0.42	0.69	-0.62	0.54
	Region N: Time (2016,2017)	0.71	1.47	0.48	0.63
	Region N: Time (2018,2019)	1.46	1.12	1.30	0.21
	Region S :Time (2016,2017)	0.56	1.32	0.42	0.68

Response variable	Explanatory	Estimate	SE	z value	p value
**Pine seedlings count (Negative binomial GLM)**	Intercept	7.29	0.18	41.38	**<0.001**
	FWI	-0.63	0.18	-3.53	**0.001**
**Deciduous seedlings count (Negative binomial GLM)**	Intercept	6.59	0.46	14.48	**<0.001**
	Region N	-0.26	0.49	-0.53	0.59
	Region S	1.66	0.53	3.12	**0.002**
	Time (2016,2017)	0.16	0.58	0.28	0.78
	Time (2018,2019)	-0.07	0.51	-0.13	0.89
**Deciduous seedlings count N=29 (zero-inflated negative binomial)**	Intercept (count model)	6.64	0.17	38.51	**<0.001**
	DMC (count model)	0.81	0.30	2.69	**0.007**
	Spruce proportion (count model)	0.68	0.21	3.24	**0.001**
	DMC: Spruce proportion (count model)	-0.61	0.16	-3.23	**0.001**
	Intercept (zero-inflation model)	-4.18	1-86	-2.25	**0.02**
	DMC (zero-inflation model)	-4.54	2.52	-1.80	0.07
	Spruce proportion (zero-inflation model)	0.08	0.63	0.13	0.89
**Deciduous seedlings count N=31 (Negative binomial GLM)**	Intercept	7.29	0.43	17.08	**<0.001**
	DMC	0.74	0.65	1.13	0.27
	Spruce proportion	0.38	0.43	0.88	0.38
	DMC: Spruce proportion	-0.48	0.46	-1.05	0.30

Response variable	Explanatory	Estimate	SE	z value	p value
**Deadwood volume (Guassian LM)**	Intercept	3.80	0.54	7.07	**<0.001**
	Region N	1.06	0.93	1.14	0.27
	Region S	0.87	1.20	0.73	0.47
	Time (2016,2017)	-1.23	0.69	-1.78	**0.08**
	Time (2018,2019)	-0.59	0.69	-0.86	0.39
	Region N: Time (2016,2017)	-1.68	1.49	-1.13	0.27
	Region N: Time (2018,2019)	-0.81	1.14	-0.72	0.48
	Region S :Time (2016,2017)	0.35	1.34	0.26	0.79
**Deadwood volume (Guassian LM)**	Intercept	2.75	0.28	9.79	**<0.001**
	Spruce proportion	0.05	0.01	3.46	**0.002**
**Deadwood volume (Guassian LM)**	Intercept	1.69	0.92	1.83	0.07
	Basal area before	0.07	0.04	1.99	**0.05**
**Deadwood volume (Guassian LM)**	Intercept	3.44	0.18	18.42	**<0.001**
	DC	0.52	0.18	2.77	**0.009**
**Deadwood volume (Guassian LM)**	Intercept	3.44	0.19	17.96	**<0.001**
	FWI	0.47	0.19	2.41	**0.02**
**Fire scar count (Negative binomial GLM)**	Intercept	6.82	0.50	13.49	**<0.001**
	Pine DBH before	-0.08	0.02	-3.39	**0.002**

Table S5. Model coefficients for the Tukey post-hoc pairwise comparisons found in the results section of the main document. P-values < 0.05 are in bold.

**Model**	**Pairwise comparison**	**Estimate**	**SE**	**z value**	**p value**
**Basal area change ~ Region**	Central- North	-0.16	0.06	-2.42	**0.04**
	Central- South	-0.17	0.06	-2.82	**0.01**
	South- North	-0.02	0.09	-0.23	0.97
**Pine seedlings ~ Region**	Central- North	1.98	0.39	5.08	**<0.001**
	Central- South	0.82	0.41	2.01	0.11
	South- North	-1.16	0.47	-2.49	**0.03**
**Deciduous seedlings ~ Region**	Central- North	0.15	0.66	0.22	0.97
	Central- South	-1.84	0.66	-2.77	**0.02**
	South- North	-1.98	0.76	-2.59	**0.03**

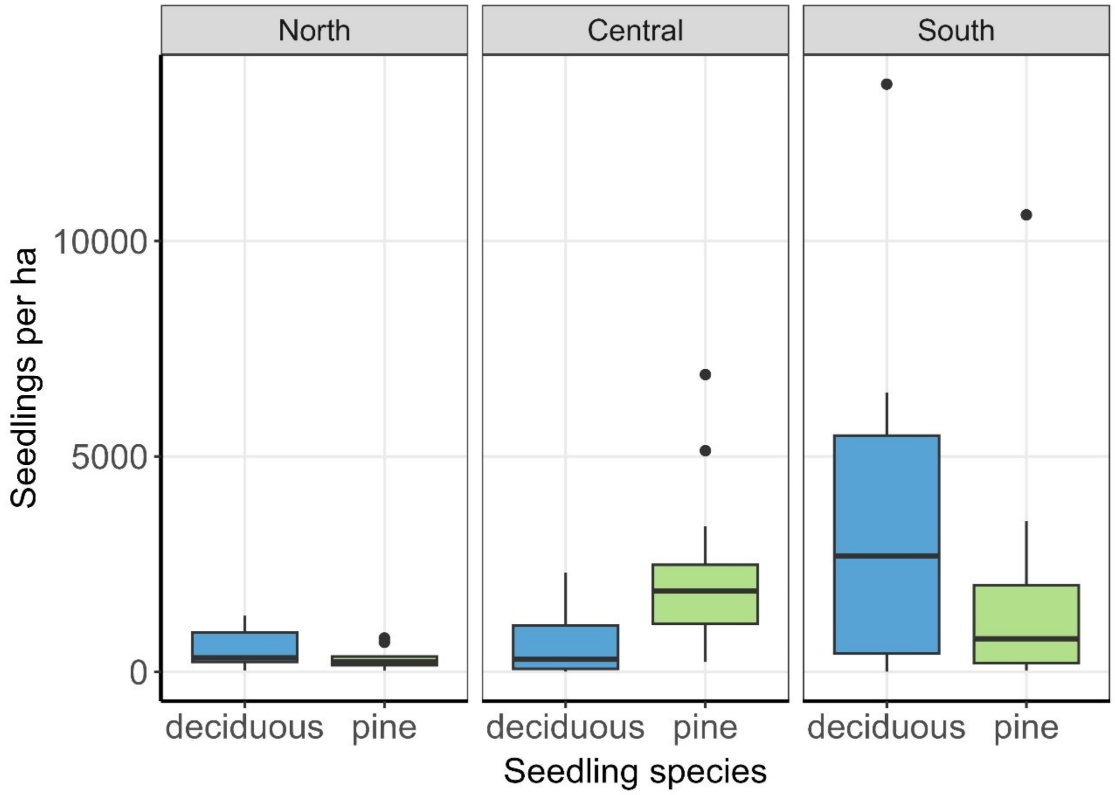

Figure S1. Boxplots of the mean number of seedlings of deciduous species (birch, aspen, rowan and sallow) and pine found per site (n=32) in the three regions included in the study: north (8), central (16) and south (8).

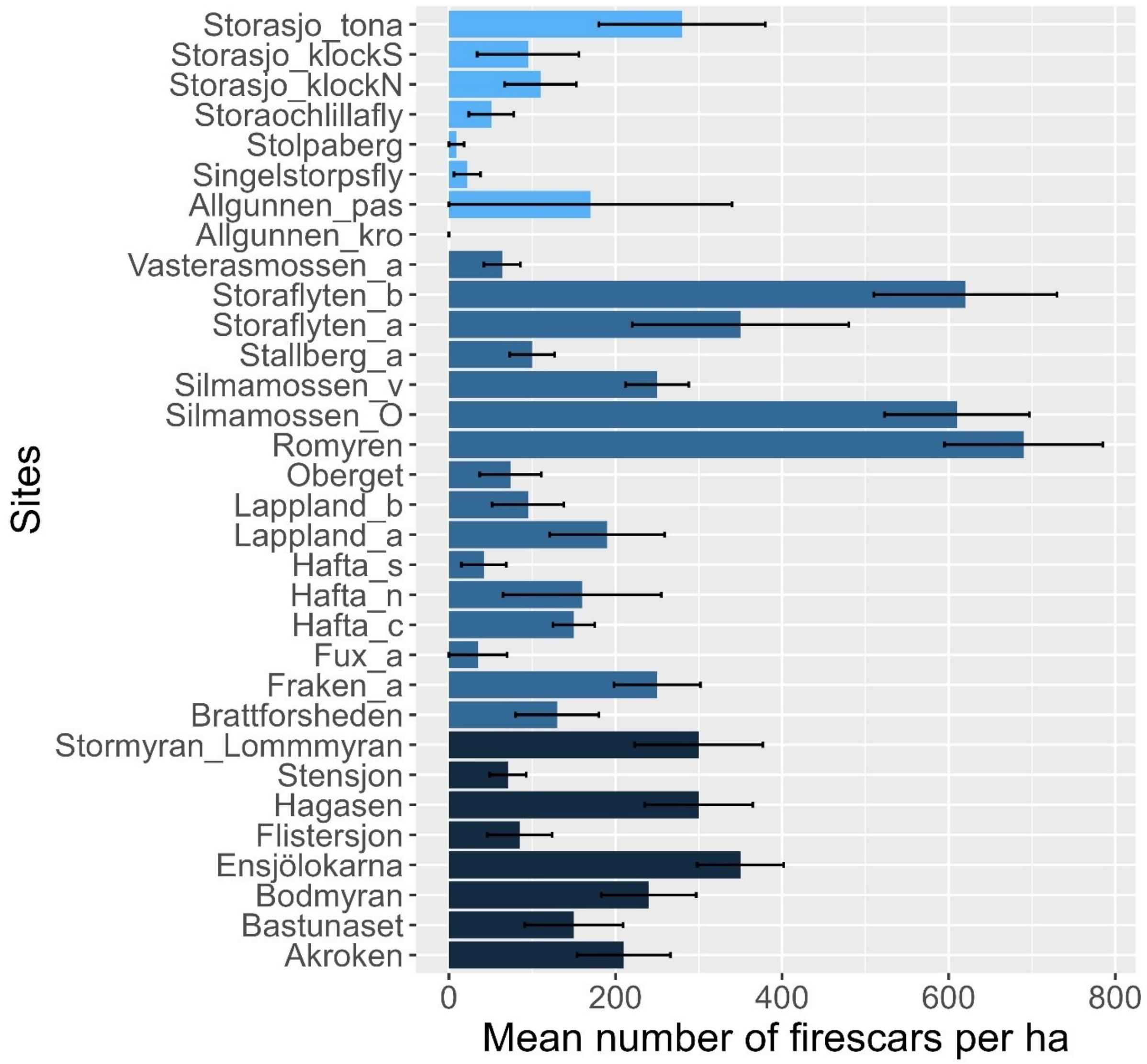

Figure S2. Bar chart showing the mean and ± se (n=32) of the number of potential fire scars per hectare and site. Sites are arranged according to region with South sites at the top of the figure (8), followed by central (16) and finally North at the bottom (8).

References

Boby, L.A., Fawcett, J.E., Clabo, D., Harriman, H., Maggard, A., Coulliette, B., Kays, L. & McNair, S. (2023). *Guidebook for Prescribed Burning in the Southern Region — Southern Regional Extension Forestry*. (WSFNR-23-16A). University of Georgia Cooperative Extension Bulletin 1560. UGA Warnell School of Forestry & Natrual Resources Outreach Publication. https://sref.info/resources/publications/guidebook-for-prescribed-burning-in-the-southern-region?fbclid=IwAR3stvNOFXofS78BOb6lz-BfNCFkfNyvHd4cOK9rW0wAozttSrFST_ueqBU [2023-08-30]

Granström, A. (2005). *Skogsbrand : brandbeteende och tolkning av brandriskindex*. Räddningsverket.

Granström, A. & Schimmel, J. (1998). *Utvärdering av det kanadensiska brandrisksystemet*. Räddnigsverket.

Van Wagner, C.E. (1987). *Development and structure of the Canadian Forest Fire Weather Index System*. Minister of Supply and Services Canada. (Forestry technical report; 35)
